# Notes on the Treatment of Subtertian Cerebral Malaria with Quinine and Galyl

**Published:** 1919-01

**Authors:** 


***Notes on the Treatment of Subtertian Cerebral Malaria with Quinine and Galyl.*** By A. W. Falconer and A. G. Anderson. *Journal of the Royal Army Medical Corps,* July, 1918.

In the attempt to cope with the various types of malaria, quinine has given an effective therapy, yet the death rate is still high enough to necessi tate search for an improvement of the method.

Cases from the Salonika force were treated with quinine, to which was added a small dose of galyl, usually 0.4 gramme, intravenously. After injection there was always a general and very marked improvement. The temperature fell, and consciousness and speech were restored.

Subtertian rings and crescents yielded to this treatment completely after two or three injections, though some of the cases seemed almost moribund before the injection.

That galyl is adequate enough in itself is doubtful. On the contrary, quinine must not be discarded, nor even diminished in quantity, but it remains a fact that soldiers who had been treated with quinine sulphate or quinine bihydrochloride exhibited immediately great improvement after 0.4 gramme galyl. For patients who do not support quinine well, it is also a valuable asset, as was proved in one of the cases.

Though experience is too limited to permit any dogmatic statement, yet it is safe to affirm that the treatment in itself is free from danger.

